# Current treatment of early breast cancer: adjuvant and neoadjuvant therapy

**DOI:** 10.12688/f1000research.4340.1

**Published:** 2014-08-19

**Authors:** Elizabeth Miller, Hee Jin Lee, Amriti Lulla, Liz Hernandez, Prashanth Gokare, Bora Lim

**Affiliations:** 1 Penn State College, State College, PA, 16801, USA; 2 Department of Pathology, University of Ulsan College of Medicine, Asan Medical Center, Seoul, 138-736, Korea, South; 3 Department of Hematology/Oncology, Penn State College of Medicine, Penn State Hershey Cancer Institute, Penn State Hershey Medical Center, Hershey, PA, 17033, USA

## Abstract

Breast cancer is the most commonly diagnosed cancer in women. The latest world cancer statistics calculated by the International Agency for Research on Cancer (IARC) revealed that 1,677,000 women were diagnosed with breast cancer in 2012 and 577,000 died. The TNM classification of malignant tumor (TNM) is the most commonly used staging system for breast cancer. Breast cancer is a group of very heterogeneous diseases. The molecular subtype of breast cancer carries important predictive and prognostic values, and thus has been incorporated in the basic initial process of breast cancer assessment/diagnosis. Molecular subtypes of breast cancers are divided into human epidermal growth factor receptor 2 positive (HER2 +), hormone receptor positive (estrogen or progesterone +), both positive, and triple negative breast cancer. By virtue of early detection via mammogram, the majority of breast cancers in developed parts of world are diagnosed in the early stage of the disease. Early stage breast cancers can be completely resected by surgery. Over time however, the disease may come back even after complete resection, which has prompted the development of an adjuvant therapy. Surgery followed by adjuvant treatment has been the gold standard for breast cancer treatment for a long time. More recently, neoadjuvant treatment has been recognized as an important strategy in biomarker and target evaluation. It is clinically indicated for patients with large tumor size, high nodal involvement, an inflammatory component, or for those wish to preserve remnant breast tissue. Here we review the most up to date conventional and developing treatments for different subtypes of early stage breast cancer.

## Introduction

Breast cancer is the most commonly diagnosed cancer in women. The latest world cancer statistics available from the International Agency for Research on Cancer (IARC) showed that 1,677,000 women were diagnosed with breast cancer and 577,000 women died in 2012 ^1^. Improvements in chemotherapy, surgery, lymph node evaluation and hormone receptor blocking therapy have successfully doubled the survival of breast cancer patients ^2^. The evolution of genomic research enabled the genetic and molecular profiling of cancers, which also revealed the profound complexity and heterogeneity of breast cancer ^3– 5^. Different molecular subtypes of breast cancer have various prognoses and responses to therapy ^6^. Such complexity makes it challenging for clinicians to keep abreast of new knowledge and novel. Therefore, this review gives an overview of current treatments for breast cancer. We will review treatment options based on the different stages and the molecular subtypes of breast cancer that are commonly used in the United States and Europe.

## Adjuvant treatment in hormone receptor positive breast cancer

Hormone receptor (estrogen and progesterone) positive breast cancers account for the largest portion of diagnosed breast cancers. The hormone receptor positive breast cancers constitute up to 65–75% of all breast cancers and this proportion is rising ^7^. The cells of this subtype of breast cancer are largely dependent on female hormone supply for their growth and survival ^8^. The understanding of the related biology is important in treatment design. Breast cancers that express hormone receptors (either estrogen or progesterone), but not the human epidermal growth factor receptor 2 (HER2) protein, are categorized as luminal A intrinsic subtype ^4^. Ki67, a nuclear protein that is encoded by *MIKI67* gene is a marker of proliferation, and is also critical in differentiating between A and B luminal subtypes ^9^. The luminal A subtype of breast cancer has the best prognosis amongst all subtypes, but even so, up to 20% of early stage luminal. A breast cancer patients experience breast cancer recurrence within 10 years after the completion of initial treatment without adjuvant treatment ^4^. Two main adjuvant therapy modalities are cytotoxic chemotherapy and endocrine (hormone receptor blocking) therapy. Both adjuvant treatment modalities improve disease free survival (DFS) and overall survival (OS) in hormone receptor positive breast cancer patients ^10^.

### Adjuvant cytotoxic chemotherapy

The Early Breast Cancer Trialists’ Collaborative Group (EBCTCG) compared an anthracycline-based regimen with a CMF (cyclophosphamide, methotrexate, and fluorouracil) regimen that was used more commonly in breast cancer starting from 1970 ^11^. In 2001, EBCTCG reported the collective data of the randomized trials in early breast cancer adjuvant systemic chemotherapy from 1985 to 2000 ^12^. This report not only showed the long term benefits of an adjuvant endocrine therapy, but also confirmed a 50% reduction of the overall mortality in 15 years, when hormone receptor positive breast cancer patients received adjuvant chemotherapy and tamoxifen for 5 years following surgery ^12^. EBCTCG subsequently reported the 10 year follow-up results after the initial report: when compared to the untreated group, the anthracycline-based chemotherapy group had an absolute gain of 8% in recurrence free survival, 6.5% in breast cancer mortality, and 5% in overall mortality. The CMF regimen similarly improved survival, but achieved 10.2% of absolute gain of recurrence free survival ^13^. CMF is one of the oldest poly-chemotherapy regimens developed for breast cancer. It was first introduced by Bonadonna *et al.* ^15^. CMF was initially given every month for a total of 12 months after primary breast surgery. Later, the same group compared 6 cycles versus 12 cycles of CMF, which showed no difference in both relapse free/OS in the two groups. An EBCTCG meta-analysis calculated a reduction of 6.2% in absolute breast cancer related mortality at 10 years follow-up, when using CMF adjuvant therapy, compared to no adjuvant chemotherapy ^16^.

Anthracycline was the next important agent to improve the efficacy of adjuvant chemotherapy. The NSABP B-11, B-12 trials showed the efficacy of doxorubicin (Adriamycin) in stage II breast cancer patients. The group who received doxorubicin plus melphalan and fluorouracil (PF) had significantly improved DFS and OS in 6 years, compared to those who received the same regimen without doxorubicin ^17^. NSABP B-15 compared 4 cycles of AC (Adriamycin 60mg/m ^2^ plus cyclophosphamide 600mg/m ^2^) with the conventional 6 cycles of CMF. A total of 2194 patients with positive nodes and a negative estrogen receptor were randomized into these two groups, and no difference in DFS or OS were shown between the two groups at the 10 year follow-up ^18^. The NSABP B-23 trial compared node negative, estrogen receptor negative patients randomized to 4 cycles of AC versus CMF, and again showed the same DFS and OS in both arms ^19^. NSABP B-16 compared 4 cycles of AC plus tamoxifen and tamoxifen alone as adjuvant therapy. This trial showed a 15% proportional reduction in average annual hazard, relapse, or death at 10 years follow-up, and a 25% relative risk reduction in comparison of tamoxifen only. Based on an indirect comparison, the degree of risk reduction in overall breast cancer related morbidity and mortality of anthracycline based regimen was much greater than that of CMF, which was 10% ^20^. The CALGB49907 trial was a randomized trial comparing CMF or AC chemotherapies with capecitabine as adjuvant chemotherapy for patients older than 65 with non-metastatic (stage I to IIIB) breast cancer. In this trial, the patients who received chemotherapy (either CMF or AC) had better relapse free survival at 3 years follow-up, resulted in 85% in chemotherapy group versus 68% in capecitabine arm respectively ^21^. A meta-analytical comparison of CMF versus anthracycline containing poly-chemotherapy regimen in the adjuvant setting showed that the standard 4 cycles of AC and the standard CMF were equivalent (RR 0.98, SE 0.05, 2p=0.67), but anthracycline-based regimens with substantially higher cumulative dosage than the initial standard regimen of 4 AC cycles (e.g., CAF or CEF) were significantly superior to the standard CMF ^13^. This offered the rationale for the development of an anthracycline based poly-chemotherapy in an adjuvant setting.

The second biggest game changer in breast cancer adjuvant chemotherapy was the introduction of taxane. After the efficacy of taxane was shown in advanced breast cancer, the BIG 02-98 trial incorporated docetaxel into the adjuvant setting. This trial compared sequential versus concurrent doxorubicin and docetaxel chemotherapy for lymph node positive breast cancer, showing docetaxel arms with improved survivals ^22^. BCIRG001 is an open label phase III multicenter randomized trial comparing early breast cancer with positive nodes who received TAC (docetaxel, doxorubicin, and cyclophosphamide), or FAC (fluorouracil, doxorubicin, and cyclophosphamide) 3 times a week for 6 cycles. Primary end point of the study was DFS. At a median follow-up of 10 years, TAC group had better DFS and OS compared to FAC group. Improved DFS in TAC group was not dependent on nodal status, hormone receptor or HER2 status ^23^. The Intergroup 9344 (INT 9344) trial that was led by NSABP in collaboration with the Eastern Cooperative Oncology Group ( ECOG) and South Western Oncology Group ( SWOG), that to address the question of whether adding 4 cycles of paclitaxel (T) to 4 cycles of AC would improve the clinical outcome. There was a 5% absolute improvement in DFS and a 3% in OS by adding paclitaxel (T), but not by adding cycles of adriamycin ^24^. The NSABP B-28 study compared 4 cycles of AC versus 4 AC plus 4 T. This trial also showed that adding T resulted in relative DFS improvement of 17%, but lesser degree of improvement in OS (7%) ^25^. Based on the result from the EBCTCG meta-analysis, the overall absolute reduction of recurrence by additional taxane to the anthracycline regimen was 2.8% and the reduction of mortality with recurrence was 1.3%. However this improvement was diluted when a very well dosed anthracycline-based regimen was used. The improvement in clinical outcome was sustained over a period of 5 years ^13^.

Not only the selection of chemotherapy agents but also the method of delivery is critical in the development of chemotherapy. The CALGB 9741 study compared 4 arms with different dosing schedules of AC-T. The first two arms were given a total of 4 cycles of all regimens every 3 weeks, while the second two arms received treatment every 2 weeks. A protocol-specified analysis was performed at a median follow-up of 36 months. Q 2 weekly dose-dense (dd) schedule improved the DFS and OS. However, there was no difference in either DFS or OS between the dd concurrent and the dd sequential schedule arms ^26^. Dose dense schedule is widely used as an AC-T schedule unless there are other factors. More recently, SWOG S0221 conducted a 2×2 design phase III trial comparing AC+G (filgrastim) versus dd AC in different combinations with either dd T (paclitaxel) for 6 cycles or weekly T for a total of 12 weeks. This trial showed equivalent progression free survival (PFS) in both weekly and 2 weekly T (82% in weekly versus 81% in dd PFS), suggesting that weekly paclitaxel could have the same efficacy without the patients having to receive the growth factor support ^27^. A Spanish group published the results from the GEICAM/2003-02 study, comparing FAC, and FAC followed by weekly paclitaxel for node negative high-risk patients. In this study, additional weekly paclitaxel for 8 weeks added a 2.7% improvement in PFS at 63.3 months follow-up ^28^.

### Adjuvant endocrine/hormone therapy

 There are two main categories of endocrine therapy agents: selective estrogen receptor modulators (SERMs) and aromatase inhibitors (AIs). SERMs competitively bind to estrogen receptors to interfere with DNA synthesis by recruiting co-repressors, and inhibit G0->G1 cell cycle progression ^29^. The three main drugs of this category are tamoxifen, raloxifen, and toremifene. AIs work differently. These drugs inhibit an enzyme called ‘aromatase’ that converts circulating testosterone to estradiol (E2), and androstenedione to estrone, by aromatization. Such peripheral conversion of other hormones to estradiol is the main source of estrogen in post-menopausal women ^30^. Therefore, AIs only work when the primary source of estrogen is terminated – either by the menopausal state, oophorectomy, or estrogen deprivation therapy using luteinizing-hormone-releasing hormone (LHRH) agonists. Exemestane, anastrazole and letrozole are three main drugs of this category.

Tamoxifen and its effects have been studied for over 3 decades in thousands of women, as a primary and secondary preventive therapeutics. It is estimated that 400,000 or more women are estimated to be alive as a result of tamoxifen therapy worldwide and that also due to tamoxifen, millions of women achieved extended DFS ^31, 32^. Fifteen years of adjuvant treatment review of EBCTCG concluded that tamoxifen successfully reduced the absolute rate of breast cancer recurrence in hormone receptor positive early stage breast cancer by 13% (2p<0.00001), and breast cancer related mortality by 9.1% (2p<0.00001) ^12^. A 5 year duration of adjuvant tamoxifen has been the standard of care for years, however ATLAS (Adjuvant Tamoxifen: Longer Against Shorter) trial showed the benefit of longer tamoxifen use. This trial accrued 80,000 women and randomized them to extend the tamoxifen therapy for 10 years versus stopping at 5 years as previously recommended. The extended treatment arm to 10 years had a 4% improvement in breast cancer related mortality ^33^. Since the result of this study, extended duration of endocrine therapy has been incorporated as the standard of care, as long as the patient can tolerate the treatment without side effects.

In post-menopausal women, AI is the regimen of choice, based on the improved efficacy compared to tamoxifen as shown in previous trials. The ATAC (anastrazole, tamoxifen, alone or in combination) trial compared the efficacy of anastrazole and tamoxifen for postmenopausal women in adjuvant settings. After a median follow-up of 68 months, anastrazole showed a significantly prolonged DFS compared to tamoxifen, significantly reduced distant metastases (324 vs 375; HR 0.86 and contralateral breast cancers (35 vs 59; 42% reduction) ^34^.

During the 5–10 years of endocrine therapy, a patient’s menstrual status can change from premenopausal to a menopausal state. Therefore, it is not surprising to raise the question whether the use of different endocrine adjuvant therapies in sequence could affect the clinical outcome. The MA-17 trial enrolled 5170 post-menopausal patients who had completed 5 years of adjuvant tamoxifen, and assigned them either to receive an additional 5 years of letrozole or a placebo. The DFS at 4 years follow up was 94.4% in the letrozole arm versus 89.8% in the placebo arm – representing 4.6% of absolute reduction in disease recurrence. Both distant recurrence and contralateral breast cancer incidence were lower in the additional letrozole adjuvant arm ^35^. The BIG-98 trial compared three groups – one group received letrozole for 5 years, another one received tamoxifen for 5 years, and the last group received sequential therapy. The letrozole arms were superior, but the DFS and OS of the sequential therapy were the same as using letrozole monotherapy. The outcome of tamoxifen followed by a letrozole arm was the same as for the letrozole monotherapy, but there was a trend towards a better outcome in the letrozole monotherapy arm, suggesting the superiority of letrozole as a first line endocrine therapy ^36^. Dowsett *et al.* compared two cohorts of postmenopausal patients’ data by meta-analysis. Cohort 1 patients started endocrine therapy with AI and continued to take AI, or converted therapy from tamoxifen to AI. At 5 years, the AI monotherapy resulted in an absolute 2.9% reduction in recurrence (9.6% for AI versus 12.6% for tamoxifen; 2P < .00001) and a non-significant 1.1% absolute reduction in breast cancer mortality (4.8% for AI vs 5.9% for tamoxifen; 2P = 0.1). Cohort 2 patients started endocrine therapy with tamoxifen for 2 years, then they were randomized to either continue tamoxifen or switch to AI. At 3 years from treatment divergence (which was about 5 years after the initiation of endocrine therapy), the group who converted the therapy to AI showed an absolute 3.1% recurrence and an absolute 0.7% reduction in breast cancer mortality ^37^. From these data taken together, AI is considered as the gold standard first line therapy for post-menopausal women in adjuvant endocrine therapy.

### Molecular assays to guide adjuvant therapy

Despite the proven benefits of chemotherapy in early stage hormone receptor positive breast cancer patients, it is also clear that the absolute benefits of chemotherapy are not the same across all patients. Traditionally, gender, ethnicity, pathologic stage of tumor, age, personal history and family history were considered to be the main factors that could help to measure the benefit of adjuvant chemotherapy in individual patients ^38^. Adjuvant Online! ^39^, a commonly used risk calculator, is a good example of such a traditional measure of prognosis. It has been widely studied and validated in different populations of patients ^12, 40, 41^. Over time, we have learned that the biologic characteristics of tumors can be more critical in adjuvant treatment decision making. Several comprehensive genomic profiling tools to characterize and predict the prognosis of individual patients have been developed. Such genomic profiling tools not only provide sub-typing of breast cancers, but also can predict their response to adjuvant therapy. For instance, Oncotype DX™ calculates the prognosis of individual patient’s 10 years recurrence risk by assessing 16 genes that are related to the proliferation of the tumor. Intriguingly, this tool also gives a validated prediction as to whether the individual patient who receives adjuvant endocrine treatment tamoxifen will have an additional benefit by the addition of chemotherapy. The predictive value of Oncotype DX™ was validated in both pre- and post-menopausal women ^42, 43^. However, there were questions remaining for patients with an intermediate score from Oncotype DX™ recurrence score testing. TAILORx (the Trial Assigning IndividuaLized Options for Treatment : NCT00310180) is being conducted to answer this question ^44^.

The application of recurrence score has been expanded to node positive patients as well. A retrospective ancillary study that analyzed tumor samples from the SWOG-8814 trial node positive breast cancer patients confirmed that patients with low Oncotype DX™ did not gain additional benefits from chemotherapy , also in some patients with positive lymph nodes. For patients with a high recurrence score on the other hand, there was significant improvement of progression free survival independent of the number of positive nodes (hazard ratio 0.59; 95% CI=0.35–1.01) ^45^. SWOG S-1007 RxPONDER (Rx for Positive Node, Endocrine Responsive Breast Cancer) ^46^, a prospective, randomized trial is currently ongoing to further determine the effect of chemotherapy for patients with up to 3 positive lymph nodes involvement. PAM50 is a more comprehensive genomic profiling tool for breast cancer. This test not only can detect the intrinsic subtype of breast cancer, but also will predict the prognosis of individual patients ^47^. However, so far the studies have not been able to validate the predictive value of PAM50 for specific therapeutic use ^48^, thus, to date, it is mainly used for prognosis/sub-typing reasons. High risk diseases that were detected via the PAM50 test are mainly non-luminal A or B cancers, the majority of them being triple negative breast cancers. MammaPrint ^49^ is another available genetic signature mainly currently used in Europe using microarray chip technology. This tool analyzes a total of 70 genes that represent 6 hallmarks of cancer. The test is also currently developed as a predictive marker for better selection of tailored therapies for breast cancer patients.

### Bisphosphonate in adjuvant therapy

Traditionally, bisphosphonate was used to treat hypercalcemia and osteoporosis as it blocks the activity of osteoclasts. Because of the relationship between cancer cells and osteoclasts in the bone marrow niche acting as a feedback loop in an interconnected microenvironment, bisphosphonate also has a great activity against bony metastasis in solid cancers. Moreover, osteoclast secreted RANKL (receptor activator of nuclear factor kappa-B ligand) and RANK combination promotes the proliferation and survival of breast cancer stem cells in pre-clinical studies, suggesting a strong scientific rationale to use inhibitors of the osteoclast activity to improve survival ^50^. However, the results from various small trials were mixed, and resulted in confusion and debate in the field. The AZURE study was the first trial to bring interest and attention towards bisphosphonate in the field by showing the survival benefits. The OS of the zolendronic acid adjuvant treatment group and a control group were 85.4% versus 83.1% respectively, with a confidence interval of 1 ^51^. NSABP B-34 ^52^, GAIN ^53^ and NATAN ^54^ trials did not show the same superiority in an adjuvant bisphosphonate use group. The actual agents used in the different trials varied – both IV and oral agents were used. Interestingly, when subgroup analysis was performed either by age 55 or menopausal status, there was improved hazard ratio in DFS in elderly, post-menopausal women. The p-values in later 3 trials were not statistically significant. Recently, large meta-analysis done by a group in United Kingdom collectively analyzed total of 18000 women from 41 different studies ^55^. The results among pre-menopausal women did not show any difference between the bisphosphonate group and non-bisphosphonate group in both recurrence free survival and breast cancer related mortality. However in menopausal women, the breast cancer related mortality was reduced by 3.1%, and the distant recurrence rate was reduced by 3.5%. Given the low side effect profile of the drug, this result will likely change standard practice in the near future.

## Adjuvant therapy in HER2 positive breast cancer

About 20–25% of breast cancers are characterized by the over-expression of HER2 protein ^56^. HER2 (ErbB2) is a transmembrane glycoprotein that has both an intracellular receptor tyrosine kinase (TK) domain and an extracellular ligand binding domain. The HER (ErbB) family consists of HER1 (ErbB1 = EGFR), 2, 3, and 4 ^57^. Different subtypes of HER protein share similar intracellular TK domains, but express distinct ligand binding extracellular domains ^56^. HER receptors are activated via homodimerization, or heterodimerization with its family member HER1 and HER3. HER2 overexpression is one of the most important carcinogenic features, as well as being a prognostic and predictive marker for response to HER2 targeted therapy ^56^. Trastuzumab is the first monoclonal antibody developed as an anti-HER2 therapeutic that binds to the juxtamembrane domain of HER2 receptor ^58^. Trastuzumab has other interesting activities; it induces the activity of p21 or p27, which then cause transcription inhibition and also induces antibody-dependent cell – mediated cytotoxicity (ADCC) ^59^. Since the first US Food and Drug Administration approval in 1997, trastuzumab has become a cornerstone of HER2 overexpressing breast cancer treatment in any stage of disease, including the adjuvant setting.

### Trastuzumab and cytotoxic therapy as adjuvant therapy

The BCIRG 006 trial accrued early stage HER2 overexpressing breast cancer patients between April 2001 and March 2004 and compared three arms: AC-T (adriamycin, cyclophosphamide, and paclitaxel), AC-TH (adriamycin, cyclophosphamide, paclitaxel and trastuzumab), and TCH (docetaxel, cyclophosphamide, and trastuzumab). The primary endpoint of DFS after a median follow-up of 65 months was 75% in the AC-T arm, 84% in the AC-TH arm, and 81% in the TCH arm. The first planned interim analysis was performed in 2006. TCH had a better side effect profile, and without a non-statistically significant difference in efficacy this led to its approval by the FDA ^60^. NCCTG (North Central Cancer Treatment Group) N9831, NSABP (National Surgical Adjuvant Breast and Bowel Project) B-31, FinHER (Finland Herceptin), HERA, NOAH (Neo-adjuvant Herceptin), FNCLCC-PACS (Federation Nationale des Centres de Lutte Contre le Cancer-Programmes d’Actions Concertees Sein) 04, BCIRG (Breast Cancer International Research Group) 006 trials all showed that a trastuzumab – chemotherapy combination regimen - leads to improved clinical outcome compared to conventional cytotoxic adjuvant therapy ^61– 65^.

The next question to address is the duration of adjuvant treatment. The HERA trial was an open label, large randomized phase III trial comparing 2 years versus 1 year use of adjuvant trastuzumab for patients with HER2 positive breast cancer. A total of 5102 patients were randomized into two groups, after completion of 1 year adjuvant trastuzumab to either stop at year point, versus 1 additional year to complete 2 years. The primary end point of this study was a PFS, and there was no difference between two groups ^62, 66^. Therefore, a year (52 weeks) of adjuvant trastuzumab treatment after surgery is currently the standard of care for early stage HER2 positive breast cancers.

However, resistance to trastuzumab therapy still remains a challenge in the treatment of HER2 overexpressing breast cancer. HER1 or HER3 can bind to the ligand, and can activate the intracellular downstream signaling of cancer cells regardless of HER2 blockage therapy. The other common mechanisms of resistance to trastuzumab include the truncated form of HER2. If the HER2 protein lacks the antibody binding domain (the truncated form of HER2 is also called p95) ^67^, it is resistant to trastuzumab due to lack of an appropriate binding site. The phosphatase and tensin homolog (PTEN) gene mutation, resulting in PTEN constant activation, can bypass the blockage of HER2-mediated intracellular signaling ^68^, insulin-like growth factor 1 receptor (IGF-R), and phosphoinositide 3-kinase (PI3KA)/Akt pathway amplification ^69^. Defective apoptosis pathways are main causes of trastuzumab resistance. Most recently, immunologic factors - different expression of stromal tumor infiltrating lymphocytes ^70^, defective Fc receptors ^71^ that can interfere with normal immune responses to trastuzumab have also been suggested as mechanisms of resistance.

### Less effective small molecules in the adjuvant setting

 Lapatinib was the first small molecule that was developed to overcome trastuzumab resistance. Lapatinib not only inhibits HER2 but also inhibits HER1 (=EGFR), although a later preclincal study ^72^ suggested that the activity of lapatinib was HER1 independent. It binds to the intracellular domain of HER2 protein, thus the efficacy is preserved for the truncated form of HER2 protein. This drug shows efficacy as a single agent, in combination with capecitabine, and with trastuzumab in metastatic settings ^73^. Unfortunately, lapatinib failed to show the efficacy in an adjuvant setting. A total of 3161 women who may have received adjuvant therapy without trastuzumab were divided into lapatinib and placebo group. DFS at 47.4 months follow up showed 13% in lapatinib versus 17% in placebo group. However this study included patients who had no HER2 protein expression by central review. The authors reported that lapatinib had a marginal benefit in women with confirmed HER2 positive breast cancer, but this may suggest inconclusive benefits of lapatinib in adjuvant settings ^74^. TD-M (emtansine-trastuzumab conjugate), and pertuzumab are newer agents targeting HER2 overexpression in breast cancer, and currently approved for use in metastatic settings by the US FDA ^75^. Pertuzumab has also been approved in neoadjuvant settings. These two agents will be discussed in the sections below.

## Adjuvant therapy for triple negative breast cancer

Triple negative breast cancer (TNBC) is a subtype of breast cancer that accounts for 10–15% of breast cancer cases. TNBC is a heterogeneous group of tumors that commonly occur in younger women, African Americans, and in BRCA gene-mutated populations ^76, 77^. It is called ‘triple negative’ because this subtype of breast cancers are negative for ER/PR/HER2 ^76^. The survival of patients with metastatic or recurrent TNBC remains poor to date, due to lack of meaningful biologic targets, and the recurrence rate is higher than other subtypes of breast cancers when compared at same stage of disease ^78^. The benefit of an adjuvant chemotherapy in TNBC is greater than in hormone receptor positive breast cancer patients, based on the data from a large meta-analysis by EBCTCG ^79^. Given the lack of effective targets in this subtype of breast cancers, chemotherapy remains as mainstay of adjuvant therapy for TNBC.

### Cytotoxic therapy

Standard regimens currently used in TNBC subgroup are the same as for hormone receptor positive cancers, since this subgroup of tumors responds well to both anthracycline or taxane based regimen ^80, 81^. The benefit of an adjuvant cytotoxic therapy is much greater in TNBC. A retrospective analysis of three large CALGB trials including 6,444 patients confirmed the substantially larger benefits of adjuvant chemotherapies for hormone receptor negative breast cancers. When comparing two different chemotherapy regimens as adjuvant treatments – CAF (cyclophosphamide, adriamycin, 5-FU) with dose dense Q2 weekly AC-T from different CALGB trials, there were a 55% relative reduction and a 28% absolute reduction of recurrent risk for hormone receptor negative tumors ^26^. Thus, TNBC patients with a tumor size greater than 1cm, or any lymph node involvement, receive cytotoxic chemotherapy unless they have significant contraindications.

One sub-group of patients among the patients with TNBC, treated with a more promising targeted therapy currently under development, are the patients with BRCA1 and 2 mutations. BRCA1 and 2 are important DNA repair genes, thus the tumorigenesis in this proportion of TNBC subtypes is higher in this population. About 10% of women with TNBC who had a cancer diagnosis at less than age 40 are found to have BRCA1 or 2 mutations ^82^. Women with TNBC younger than 50 years old could present up to 10–25% BRCA1/2 mutation incidence ^83^, which offers a good rationale for using platinum agents. In addition, more than half of TNBCs have mutation of TP53 ^47^, which gives another rationale of platinum sensitivity, given the pre-clinical data from breast cancer cell studies showing that cells are more sensitive to platinum agents when they have a defect/mutation in the p53 family proteins. Currently, the platinum agents – mainly carboplatin – are studied in neoadjuvant settings where the study outcome can be assessed in a short period of time. However the use of platinum agents in adjuvant setting is continuously evolving in this subgroup of breast cancers.

### Anti-angiogenesis agents

Pre-clinical studies and early phase clinical studies revealed the importance of angiogenesis and microenvironment in triple negative breast cancer cells, suggesting the efficacy of VEGF targeted therapy ^84, 85^. Based on exciting early data, the BEATRICE trial enrolled total 2591 patients with early stage breast cancer, divided them into two groups – one group to receive standard adjuvant chemotherapy and monitor, and the other to receive standard chemotherapy (either anthracycline or taxane based on investigator’s choice) + 5mg/kg weekly equivalent bevacizumab, and followed by bevacizumab maintenance. Unfortunately, 3-year DFS was 82.7% in the chemotherapy only group versus 83.7% in the bevacizumab group. There was a certain trend towards bevacizumab benefits in patients who had a high pre-treatment plasma VEGFR-2 level, but this was not statistically significant, disappointing researchers and clinicians in the field ^86^.

## Neoadjuvant therapy

Traditional indications for neoadjuvant therapy in breast cancer include N2 stage - fixed or matted lymph node on ipsilateral side, or clinically apparent ipsilateral internal mammary nodes in the absence of axillary node, making the clinical staging at least stage IIIA or above. Patients with stage IIIB disease with tumors invading the chest wall, skin or both, or with breast cancer of inflammatory nature, would be a good candidate for neoadjuvant therapy ^87^. Neoadjuvant therapy should also be considered for women with clinical stage IIA and IIB tumors with a larger tumor who wish to have breast-conserving operations and avoid mastectomy. Not in all, but in many patients, neoadjuvant therapy results in sufficient tumor response to make breast-conserving operations possible. Several studies in the early 2000s showed that neoadjuvant chemotherapy successfully reduced both locoregional and in breast tumor recurrence even in large T3 and T4 tumors ^88, 89^. More recently, pathologic complete remission (pCR) is an important concept currently developed as a prognostic marker of survival in breast cancer patients that can be used as a surrogate outcome of survival ^53, 90^. Neoadjuvant therapy has been evolving rapidly given this benefit ^91^.

## Neoadjuvant therapy for hormone receptor positive breast cancer

There have been many trials comparing the clinical outcome of pre-operative versus post-operative therapy. The EORTC10902 trial accrued 698 patients early stage breast cancer (both hormone receptor positive and negative) randomized to 4 cycles of 5-FU, epirubicin, and cyclophosphamide (FEC) administered pre-operatively versus the same regimen given post-operatively. PFS, OS, or local recurrence rate were not different when comparing pre-operative and post-operative therapy ^92^.

From the meta-analysis, Mauri and colleagues found no difference with regard to death (RR 1.00, 95% CI, 0.90 to 1.12), disease progression (RR 0.99, 95% CI, 0.91 to 1.07), or distant disease recurrence (RR 0.94, 95% CI, 0.83 to 1.06). However, the rate of local recurrence was higher in the neoadjuvant group (RR 1.22, 95% CI, 1.04 to 1.43). This was mainly in trials where surgery was avoided in cases of clinical complete response ^93^.

Nonetheless, for an individual patient, the delay of surgery by pre-operative therapy could provide potential harm. Given that all randomized trials are comparisons of cohorts, the disadvantages of single patients are not reflected in the overall results. Fortunately, the proportion of tumors progressing during neoadjuvant therapy is very low, but hypothetically even if the tumor as a whole is shrinking, single tumor cells could respond differentially. As discussed previously, partly resistant tumor cells might acquire full-blown resistance during neoadjuvant treatment and generate micrometastases.

### Neoadjuvant hormonal therapy

A broad use of endocrine therapy as a tool in neoadjuvant settings could be somewhat limited due to the slow response rate of tumors in general, requiring long duration of therapy and risking the benefit of early surgical intervention ^94^. Also, other important benefits of using a neoadjuvant therapy – to assess the response of tumor to the treatment, to explore the prediction of long term relapse free survival - are less obvious in hormone receptor positive breast cancers ^91^. Thus, an endocrine therapy as a neoadjuvant therapy tool has been tested primarily in postmenopausal women who aimed to change the extent of surgical interventions from a mastectomy to a breast-conserving operation, but who were not fit for chemotherapy due to medical co-morbidities. When the response rates were compared between AI and tamoxifen in this setting, the clinical response rate was significantly higher in the AI group than in the tamoxifen group, but overall the pCR was less than 10% ^95^. To date, there has not been a direct comparison of long term progression free, or overall survival between neoadjuvant endocrine therapy followed by surgery versus surgery followed by adjuvant therapy.

A study conducted by Cameron *et al.* compared endocrine neoadjuvant therapy only group with a group who received both endocrine therapy and cytotoxic therapy, after being found not to have significant response in operable breast cancer patients, and two groups did not show a difference in clinical outcome ^96^. The number of axillary lymph nodes after systemic chemotherapy, and the rate of response after either endocrine or pre-operative chemotherapy were the factors predicting survival outcome, the number of lymph nodes more strongly so, suggesting that as long as the patient responds to hormone targeted therapy, the prognosis is good despite the overall low response rate ^97^.

When Semiglazov *et al.* compared anastrozole to chemotherapy in elderly women in, both had equivalent benefit when used in a neoadjuvant setting for women older than 70, with hormone receptor positive cancer ^98^. In the IMPACT trial which compared anastrozole, tamoxifen, and a combination of both agents in postmenopausal women, three arms showed similar response rate. However, for women who needed mastectomy at baseline, anastrozole showed a significant improvement in terms of downgrading the extent of surgery from a mastectomy to a breast-conserving operation ^99^. There is currently not much information available on the use of neoadjuvant endocrine treatment in premenopausal patients.

A phase II study was conducted to see whether everolimus added to letrozole for operable breast cancer patients would improve the clinical response. Everolimus is an inhibitor of mTOR, a downstream signaling molecule of Akt. A group who received the everolimus combination had higher response compared to the letrozole and placebo group (68% versus 59.1%). The everolimus combination arm also showed a reduction in the expression of the biomarker Ki67, as well as a phosphor-S6, in surgical specimens, suggesting downregulation of the proliferation and PI3K pathway. The safety profile of the everolimus arm was similar to monotherapy with everolimus ^100^. These results suggest that an including an additional mTOR inhibitor with the hormone therapy may improve the overall response rate in neoadjuvant settings. However the potential benefit on survival rate is still unclear, hence it needs to be further investigated.

### Neoadjuvant cytotoxic therapy for hormone receptor positive cancer

Despite the lower response rate of hormone receptor positive breast cancer patients to chemotherapy, compared to patients with other subtypes of cancer, chemotherapy is still the main neoadjuvant systemic therapy that has been widely studied and used ^101, 102^. So far, molecular profiling to predict the benefits of using different options of therapy in the neoadjuvant setting have not been well studied. Therefore, there is no good prediction tool to select the perfect candidate of neoadjuvant chemotherapy among hormone receptor positive breast cancer patients. Because of this reason, in the treatment of operable hormone receptor positive breast cancer patients, a more personalized neoadjuvant systemic cytotoxic therapy based on the patient’s wish and clinical scenario is preferred rather than a standardized therapy.

Anthracycline/taxane-based chemotherapy regimens have been studied extensively in prospective randomized trials. Overall, pCR is between 15% and 20% in hormone receptor positive breast cancer patients who are pre-operatively treated with cytotoxic therapy. The main regimens studied in neoadjuvant settings include AC followed by docetaxel or paclitaxel, epirubicin/paclitaxel-CMF, and a dose-dense sequence of epirubicin and paclitaxel ^103– 106^.

## Neoadjuvant therapy for HER2 overexpressing breast cancer

HER2 overexpression is a good predictive marker of HER2 targeted therapy, which means that HER2 therapy will be very effective in reducing the size of HER2 positive breast cancers. Therefore, the size of HER2 positive breast cancer can easily be reduced in patients who wish to have a breast-conserving operation, and potentially improve the outcome of patients if pCR can be achieved. pCR in HER2 overexpressing breast cancer after neoadjuvant therapy is associated with improved survival.

### HER2 targeted agents in combination with cytotoxic therapy

A randomized phase II study (CHER-LOB) showed that the combination of lapatinib and trastuzumab is superior in achieving breast-conserving surgery or pCR in HER2 positive breast cancer patients, compared to either trastuzumab or lapatinib alone in combination with 12 weeks of paclitaxel followed by FEC chemotherapy. The rates of breast-conserving surgery were 66.7%, 57.9%, and 68.9% in trastuzumab alone (arm A), lapatinib alone (arm B) and combination arm (arm C), respectively. The pCR rates were 25% (90% CI, 13.1% to 36.9%) in arm A, 26.3% (90% CI, 14.5% to 38.1%) in arm B, and 46.7% (90% CI, 34.4% to 58.9%) in arm C (exploratory *P* = .019), showing improved efficacy in double targeting arm ^107^. The TECHNO trial also evaluated pre-operative EC (epirubicin+cyclophosphamide) followed by TH (paclitaxel+trastuzumab) in HER2 overexpressing breast cancer. The DFS of patients who achieved pCR was 88% compared to patients without pCR 73% (p=0.01). pCR was the only significant prognostic factor for DFS (HR 2.5; 95% CI 1.2 to 5.1; p=0.013) from multivariate analysis. Patients who did not achieve pCR had an increased risk for relapse and death ^108^. NSABP B-41 trial studied single agent lapatinib combined with ACT, in comparison with dual HER2 blockade with lapatinib and trastuzumab and ACT. pCR was achieved in 52.5% of the patients in the trastuzumab arm versus 53.2% in the lapatinib arm, compared to 62% in the treatment arm with combination of trastuzumab and lapatinib, thus showing a significant improvement by using double targeting therapy to achieve pCR ^109^.

Pertuzumab is a recombinant humanized monoclonal antibody that targets the extracellular dimerization domain (sub-domain II) of HER2, as well as binding to the ligand binding site of HER3 ^110^. To date, there have been 2 neoadjuvant trials to test the role of trastuzumab – Neosphere and Tryphaena ^111, 112^. The NeoSphere trial studied combination of dual HER2 blockade – with ertuzumab, trastuzumab, and docetaxel givenery 3 weeks for a total of 4 cycles. Following surgery, all patients received 3 cycles of FEC IV every 3 weeks and trastuzumab was administered IV every 3 weeks to complete 1 year of therapy. The trial’s primary endpoint was a pCR rate defined as the absence of invasive cancer in the breast (ypT0/is). The addition of pertuzumab resulted in increased rate of pCR, 45.8% [95% CI 36.1–55.7] compared from 29.0% [95% CI 20.6–38.5] ^111^. Based on improved pCR rate, the US FDA approved the use of pertuzumab in combination with trastuzumab and docetaxel for both metastatic and neoadjuvant setting of HER2 positive breast cancers ^113^.

## Neoadjuvant therapy for triple negative breast cancer patients

The role of neoadjuvants in TNBC subtype cancers is somewhat mixed, and non-linear. From an analysis of 1118 patients who received neoadjuvant chemotherapy at the MD Anderson Cancer Center for stage I-III breast cancer during 1985 to 2004, 23% (total of 255 patients) had TNBC. TNBC patients had higher pCR rates compare to non-TNBC patients, but had rather decreased 3 year progression free survival, OS, and post-recurrence survival. For patients who achieved pCR, the outcomes were similar in both groups. When patients had TNBC, the recurrence and death rates were higher in the first 3 years, and once they had recurrence, the survival was significantly worse ^81^.

### Cytotoxic neoadjuvant therapy for TNBC

The NSABP-18 trial results showed that the breast-conserving success rate was higher after neoadjuvant chemotherapy, when compared to same baseline characters but without neoadjuvant therapy. However, the trial did not result in disease specific mortality advantage for stage II tumors. The NSABP B-27 study had three arms with AC or AC and docetaxel pre-operatively, or AC followed by post-operative docetaxel. In this study, the patients who received AC and docetaxel for 4 cycles pre-operatively had higher pCR rate; however this did not result in OS or DFS benefit. The administration of docetaxel post-operatively improved DFS in patients who had a partial response to pre-operative AC. All adjuvant chemotherapy regimens are thought to be appropriate to use in neoadjuvant settings as well ^103^.

A meta-analysis observed the clinical outcome of TNBC and non-TNBC patients who received platinum-based adjuvant therapy compared to those who did not receive platinum-based therapy. The clinical complete response and pCR rate were both higher in TNBC when platinum-based chemotherapy was used ^114^. Based on accumulated data in neoadjuvant settings, platinum agents will be likely incorporated into the standard of care treatment of TNBC in the near future.

### Novel neoadjuvant therapy for TNBC

As previously mentioned, the carriers of BRCA1 and 2 mutations are susceptive of DNA breakage due to defective DNA repair machinery. Therefore, poly ADP ribose polymerase (PARP) inhibition, which could be a rescue mechanism of DNA repair when BRCA proteins are not available, creates a ‘synthetic lethality’ when given with DNA damaging agents. The I-SPY2 trial concluded that veliparib and carboplatin combination treatment arm for TNBC patients is eligible to be moved to the phase III trial, given the 90% probability of superiority, when compared to standard the chemotherapy arm ^115^. Based on this promising result, this combination arm could be considered as standard of care.

## Summary and conclusion

Breast cancer treatment has achieved the biggest strides in the improvement of survival over the last few decades. Unfortunately, many women still experience recurrence of disease, or metastasis of primary tumor after early stage tumor has been treated. A better understanding of the underlying biology of the heterogenic nature of breast cancer has already enabled the development of targeted therapy and profiling tools to reduce the disease recurrence and mortality rate caused by breast cancers. However, there are still many questions to be answered, and patients to be saved. Cooperative efforts of both basic science of discovery and development of novel strategy to target individual tumors, enhanced understanding of tumor biology, faster adaptation of novel therapy among treating health professionals, as well as novel design of clinical trials will further improve our odds in the war of breast cancer, a disease that still causes the death of 425,000 women each year world-wide.
