# Supplementary material for: Current treatment of early breast cancer: adjuvant and neoadjuvant therapy
Source: F1000Res. 2014 Aug 19;3:198. [Version 1] doi: 10.12688/f1000research.4340.1 (PMC4224200; doi:10.12688/f1000research.4340.1)
Supplement: Peer review history

# Peer review: Referee response for version 1

**DOI:** 10.5256/f1000research.4646.r6636

**Published:** 2014-11-04

**Authors:** Ahmad Awada

**Affiliations:** 1 Institut Jules Bordet, Universite Libre de Bruxelles, Brussels, Belgium

This is a very concise paper given the key messages from a wide and evolving topic. The authors provide important information and summary for clinicians.

I’m supporting the indexation of this paper.

**Suggestions**

**Title: **I propose adding: key messages from adjuvant and neoadjuvant therapy

**Abstract: **One minor comment: last sentence of the abstract: … developing early treatments for different…

I have read this submission. I believe that I have an appropriate level of expertise to confirm that it is of an acceptable scientific standard.

# Peer review: Referee response for version 1

**DOI:** 10.5256/f1000research.4646.r5867

**Published:** 2014-08-28

**Authors:** Daniel Vorobiof

**Affiliations:** 1 Department of Medical Oncology, Sandton Oncology Centre, Johannesburg, Gauteng, South Africa

This is an excellent overview on the current therapy of breast cancer, divided according to different specific stages and patients and tumor characteristics.

The abstract as well as the rest of the text provide a detailed explanation of the different clinical breast cancer stages and sub-stages and its corresponding medical treatments. The authors have reviewed the extensive relevant published literature and, when possible, referenced with the latest publications in the specific subsets.

This publication will be of value to medical students, fellows in the areas of clinical medicine and for specialists with an interest in breast cancer. It is well written and each section appropriately referenced.

I have read this submission. I believe that I have an appropriate level of expertise to confirm that it is of an acceptable scientific standard.
